# 

**Published:** 1868-10

**Authors:** 


### Special Notice.

Hereafter, "Hall's Journal of Health" will be published by J. S. Redfield, 140 Fulton St. New York, to whom all communications must be addressed in reference to the Journal or any of the Editor's books. The Editor very specially desires to be visited at his private residence by those only who wish medical advice; he will aim to be at home every day until three o'clock in the afternoon.

The publisher will not be accountable for any losses of money sent by mail; post office or bank checks payable to the order of J. S. Redfield, will always be safe.

All the publications of the Editor of this Journal will be supplied by J. S. Redfield; if sent by mail ten cents additional will be necessary. Any back number will be sent post-paid for 15cts. Any subscriber of this year who has failed to receive any number, will be supplied without charge, by mail.

